# Inflammatory Prognostic Index: A Novel Predictor of In-Stent Restenosis Following Drug-Eluting Stent–Percutaneous Coronary Intervention

**DOI:** 10.3390/diagnostics16050647

**Published:** 2026-02-24

**Authors:** Cemre Turgul, Saban Kelesoglu

**Affiliations:** 1 Department of Cardiology, University of Health Sciences, Kayseri City Training and Research Hospital, Kayseri 38 000, Turkey; cemrebarhana_@hotmail.com; 2 Department of Cardiology, Erciyes University Faculty of Medicine, Kayseri 38 000, Turkey

**Keywords:** inflammatory prognostic index, in-stent restenosis, drug-eluting stent, C-reactive protein, neutrophil-to-lymphocyte ratio, coronary artery disease

## Abstract

**Background:** The Inflammatory Prognostic Index (IPI) is a novel biomarker integrating C-reactive protein (CRP), albumin, and white blood cell-derived ratios, reflecting systemic inflammation and nutritional status. Inflammation is central to in-stent restenosis (ISR). This study investigated the prognostic value of IPI in predicting ISR after drug-eluting stent (DES) implantation. **Methods:** We retrospectively analyzed 571 patients who underwent DES implantation and follow-up angiography at a median of 12 months (IQR 12–24) for recurrent angina or ischemia. Patients were grouped as ISR (+) (*n* = 218) and ISR (−) (*n* = 353). IPI was calculated as (CRP × neutrophil-to-lymphocyte ratio)/albumin. Logistic regression and ROC analyses assessed the predictive role of IPI. **Results:** ISR occurred in 38.1% of patients. The ISR (+) group showed a higher prevalence of hypertension and active smoking, as well as higher CRP, glucose, and neutrophil levels, but lower albumin and lymphocytes (all *p* < 0.05). Elevated IPI independently predicted ISR (OR = 2.90; 95% CI = 2.35–3.57; *p* < 0.001). ROC analysis showed an optimal cutoff of 1.275 (sensitivity 84.4%, specificity 74.5%). **Conclusions:** IPI, derived from routine laboratory tests, independently predicts ISR after DES implantation and may serve as a simple, inexpensive biomarker for coronary artery disease risk stratification.

## 1. Introduction

Coronary artery disease (CAD) remains one of the leading causes of global morbidity and mortality, accounting for over nine million deaths annually worldwide [1]. Despite major advances in preventive cardiology and the widespread use of pharmacologic therapies such as statins, antiplatelets, and antihypertensives, ischemic events requiring coronary intervention are still frequently encountered [2]. Atherosclerotic coronary stenosis or thrombosis represents the fundamental mechanism underlying angina pectoris and acute myocardial infarction. Consequently, many patients with CAD undergo revascularization procedures such as percutaneous coronary intervention (PCI) or coronary artery bypass grafting (CABG) [3,4,5].

Over the past two decades, PCI has become the predominant revascularization technique for CAD [6]. Particularly in acute coronary syndromes, PCI enables rapid restoration of coronary blood flow and has become the first-line strategy regardless of lesion complexity [7]. Drug-eluting stents (DES) are now standard in PCI; however, despite ongoing improvements in stent technology and antiplatelet therapy, in-stent restenosis (ISR) remains a major limitation affecting long-term outcomes [8,9,10,11]. ISR accounts for PCI failure in approximately 10–37% of patients within three years [8,9,10,11]. While early ISR is typically related to mechanical factors such as inadequate expansion or malapposition [12,13,14,15], late ISR is mainly driven by atherosclerotic progression and inflammation-induced neointimal hyperplasia [16].

Inflammation is a key determinant in the initiation, progression, and complications of cardiovascular diseases [17]. It contributes to ISR through impaired endothelial repair, smooth muscle proliferation, and platelet activation [18,19,20,21,22,23]. Hematologic and biochemical inflammatory indices such as high-sensitivity C-reactive protein (hs-CRP), neutrophil-to-lymphocyte ratio (NLR), platelet-to-lymphocyte ratio (PLR), and systemic inflammatory index (SII) have all been linked to ISR development [19,24,25,26].

Recently, the Inflammatory Prognostic Index (IPI)—a novel composite biomarker combining the CRP/albumin ratio (CAR) and NLR—has been proposed as a comprehensive indicator of systemic inflammation and nutritional status [27,28]. Initially introduced in oncology, IPI has gained attention in cardiovascular research; however, data on its relationship with ISR are lacking. Therefore, this study aimed to investigate the association between IPI and ISR occurrence, and to evaluate the prognostic significance of IPI in predicting ISR following DES implantation.

## 2. Materials and Methods

### 2.1. Study Design and Study Population

This retrospective, single-center observational study was conducted on patients who underwent coronary angiography (CAG) at the Cardiology Department of Erciyes University Faculty of Medicine between January 2020 and January 2025 and were diagnosed with in-stent restenosis (ISR) following drug-eluting stent (DES) implantation. The indication for follow-up CAG was established in the presence of typical chest pain or evidence of myocardial ischemia detected by noninvasive stress testing (i.e., a positive exercise stress test and/or ischemia on myocardial perfusion scintigraphy).

A total of 571 patients were included in the study. All participants had previously undergone percutaneous coronary intervention (PCI) for acute coronary syndrome (ACS)—either primary or elective—with DES implantation during the procedure and underwent control CAG at an average of 12–24 months (median: 12 months, interquartile range: (IQR) 12–24 months) after the index intervention. Patients who did not experience any other coronary events or reinterventions during the follow-up period were retrospectively evaluated in terms of their clinical, demographic, laboratory, and angiographic characteristics. This study was conducted in accordance with the Declaration of Helsinki and was approved by the Erciyes University Clinical Research Ethics Committee (approval date: 19 March 2025; Approval Number: 2025/145). Written informed consent was waived due to the retrospective nature of the study.

Inclusion Criteria

- Age ≥18 years;
- History of successful PCI performed for ACS;
- At least one control CAG performed 12–24 months after DES implantation;
- The initial DES implantation having been performed in the setting of ACS;
- Availability of complete clinical, laboratory, and angiographic data.

Exclusion Criteria

Patients meeting any of the following criteria were excluded from the study:

- Presentation with stent thrombosis;
- Previous history of coronary artery bypass grafting (CABG);
- Presence of active infection, known malignancy, or hematologic disease;
- End-stage liver or renal failure;
- History of chronic inflammatory or autoimmune disease;
- Suboptimal stent implantation during the index PCI procedure;
- Balloon angioplasty alone or bare-metal stent (BMS) implantation for the culprit lesion;
- Incomplete or insufficient clinical, laboratory, or angiographic data.

### 2.2. Data Collection

Demographic characteristics (age, sex, active smoking, diabetes mellitus [DM], hypertension [HT], chronic obstructive pulmonary disease [COPD]/asthma, dyslipidemia, and history of cerebrovascular events), clinical parameters (blood pressure values, presence of heart failure, and atrial fibrillation), laboratory findings (complete blood count, biochemical parameters, lipid profile, C-reactive protein [CRP], and albumin levels), angiographic features (lesion location, stent type, and number of affected vessels), and discharge medications (antiplatelet, statin, and antihypertensive therapies for secondary prevention) of all included patients were retrospectively retrieved from patient files and/or electronic medical records.

The diagnosis of diabetes mellitus (DM) was established according to the 2019 American Diabetes Association (ADA) Standards of Medical Care, defined as fasting plasma glucose ≥126 mg/dL, 2 h OGTT plasma glucose ≥200 mg/dL, HbA1c ≥6.5%, or a prior diagnosis/use of antidiabetic treatment [28].

Hypertension (HT) was defined in accordance with the 2018 European Society of Cardiology/European Society of Hypertension (ESC/ESH) Guidelines as systolic blood pressure ≥140 mmHg and/or diastolic blood pressure ≥90 mmHg, or current use of antihypertensive medication [29].

Active smoking was defined as regular cigarette smoking for at least one year.

Dyslipidemia was diagnosed based on any of the following: low-density lipoprotein cholesterol (LDL-C) > 130 mg/dL at admission, a previous diagnosis of dyslipidemia, or current use of lipid-lowering therapy (statins, ezetimibe, etc.) [30].

### 2.3. Laboratory Analyses and Index Calculations

Venous blood samples were obtained from the antecubital vein in the morning prior to coronary angiography. Samples were immediately delivered to the laboratory for analysis.

Biochemical parameters were measured using an automated COBAS^®^ c701 system (Roche Diagnostics, Mannheim, Germany), and hematologic parameters were analyzed using a Sysmex K-1000 hematology analyzer (Sysmex Corporation, Kobe, Japan).

Inflammatory and nutritional composite indices were calculated as follows:

- NLR (Neutrophil-to-Lymphocyte Ratio) = Neutrophil count/Lymphocyte count;
- CAR (CRP-to-Albumin Ratio) = CRP (mg/L)/Albumin (g/L);
- IPI (Inflammatory Prognostic Index) = (CRP × NLR)/Albumin.

All echocardiographic examinations were performed within the first 24 h after coronary angiography using a GE Vivid E5 ultrasound system (GE Healthcare, Piscataway, NJ, USA) equipped with a 3.5-MHz transducer. Left ventricular ejection fraction (LVEF) was calculated using the biplane Simpson’s method, in accordance with the recommendations of the American Society of Echocardiography (ASE).

### 2.4. Angiographic Analysis and Stent Implantation

Coronary angiography was performed via the radial or femoral approach using the standard Judkins technique. All angiographic procedures were carried out by experienced interventional cardiologists. Patients requiring PCI were treated according to standard strategies outlined in the latest international guidelines [29].

Following the procedure, all patients received secondary prevention therapy for coronary artery disease, including dual antiplatelet therapy (DAPT)—oral aspirin (100 mg/day) combined with either clopidogrel (75 mg/day), prasugrel (10 mg/day), or ticagrelor (90 mg twice daily) for at least 12 months—along with high-intensity statin therapy, angiotensin-converting enzyme inhibitors (ACEIs) or angiotensin receptor blockers (ARBs), and beta-blockers [30].

The indication for follow-up coronary angiography was established in patients with recurrent angina pectoris or evidence of myocardial ischemia on noninvasive tests (positive exercise stress test or ischemia detected on myocardial perfusion scintigraphy). Angiographic images were analyzed for ISR according to current diagnostic criteria; luminal narrowing >50% within the stented segment or within 5 mm proximal and distal to the stent edges was defined as ISR [31].

To ensure objectivity, all angiographic images were independently reviewed by two experienced interventional cardiologists blinded to the study protocol. Any discrepancies between observers were resolved by consensus with a third senior cardiologist.

Baseline CAG images of all included patients were reviewed to confirm that all prior PCI and stent implantations had been performed in accordance with current guideline recommendations [29].

All implanted stents were verified to be second-generation drug-eluting stents (DES), either zotarolimus- or everolimus-eluting. The use of glycoprotein IIb/IIIa inhibitors during PCI was left to the discretion of the operating interventional cardiologist.

### 2.5. Statistical Analysis

All statistical analyses were performed using Turcosa Analytics v1.0.0 (Melikgazi, Kayseri, Turkey) and SPSS Statistics for Windows, Version 24.0 (IBM Corp., Armonk, NY, USA). The normality of data distribution was assessed using the Shapiro–Wilk test, and verified visually through histograms and Q–Q plots. Continuous variables were expressed as mean ± standard deviation (SD) or median (interquartile range, IQR), as appropriate according to data distribution. Non-normally distributed continuous variables were presented as median (IQR).

For comparisons between groups:

- The independent samples t-test was used for normally distributed continuous variables;
- The Mann–Whitney U test for non-normally distributed continuous variables;
- The chi-square (χ^2^) test for categorical variables.

Categorical variables were presented as number (*n*) and percentage (%). To identify variables potentially associated with coronary artery disease progression, univariate analyses were initially performed. Variables with *p* < 0.01 in univariate analysis were subsequently included in multivariate logistic regression models.

To minimize the risk of multicollinearity, inflammatory parameters were analyzed in separate multivariate models. Finally, the receiver operating characteristic (ROC) curve analysis was performed to determine the sensitivity and specificity of IPI, CAR, and NLR values in predicting coronary artery disease progression.

## 3. Results

A total of 571 patients were included in the study. Patients were divided into two groups according to the presence of in-stent restenosis (ISR): the ISR (+) group (*n* = 218) and the ISR (−) group (*n* = 353). The demographic and clinical characteristics of the participants are summarized in Table 1.

No statistically significant differences were observed between the groups regarding age or sex (*p* > 0.05). However, the prevalence of hypertension (HT) and active smoking was significantly higher in the ISR (+) group compared to the ISR (−) group [HT: 178 (50.4%) vs. 130 (59.6%), *p* = 0.032; active smoking: 57 (16.1%) vs. 52 (23.9%), *p* = 0.023]. There were no significant differences between the groups in terms of diabetes mellitus (DM), heart failure, or medication use (*p* > 0.05).

When laboratory parameters were compared, the ISR (+) group showed significantly higher neutrophil count (4.48 ± 1.09 vs. 6.39 ± 2.96, *p* < 0.001), NLR [1.62 (1.38–1.86) vs. 2.50 (2.04–3.10), *p* < 0.001], CRP (4.07 ± 3.83 vs. 6.10 ± 4.18, *p* < 0.001) and CAR [0.41 (0.24–0.81) vs. 1.26 (0.71–2.13), *p* < 0.001] values. Similarly, IPI values were significantly higher in ISR (+) patients compared to ISR (−) patients [0.71 (0.38–1.37) vs. 3.13 (1.67–6.50), *p* < 0.001] (Table 2).

The results of the multivariate logistic regression analysis performed to identify independent risk factors associated with ISR are presented in Table 3. Variables significantly associated with ISR in univariate analysis (HT, active smoking, NLR, CRP, CAR, and IPI) were included in the multivariate model. The analysis revealed that high IPI values were an independent predictor of ISR (Odds Ratio [OR]: 2.898; 95% Confidence Interval [CI]: 2.351–3.573; *p* < 0.001). Additionally, both elevated CAR (OR: 6.429; 95% CI: 4.176–9.899; *p* < 0.001) and elevated NLR (OR: 3.392; 95% CI: 2.516–4.574; *p* < 0.001) were found to be independent determinants of ISR.

**Figure 1:**
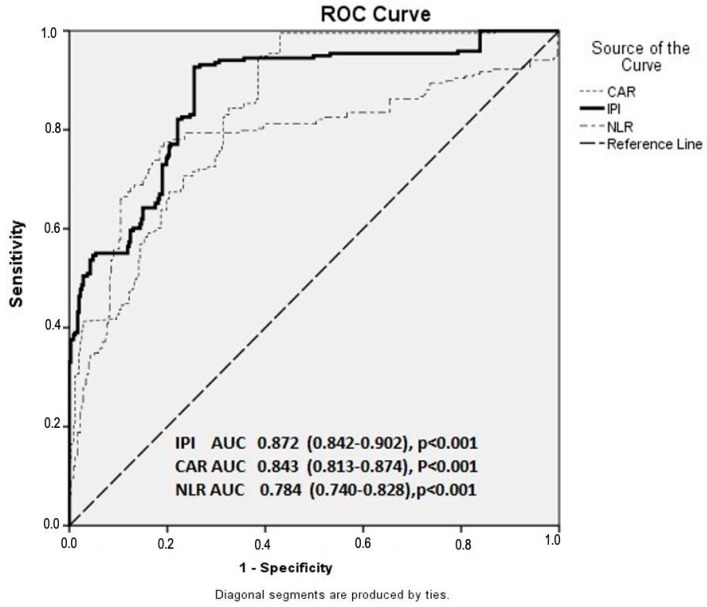
Receiver operating characteristic (ROC) curves demonstrating the predictive performance of IPI, CAR, and NLR for in-stent restenosis. Abbreviations: IPI—inflammatory prognostic index; CAR—C-reactive protein-to-albumin ratio; NLR—neutrophil-to-lymphocyte ratio; ISR—in-stent restenosis.

In the ROC curve analysis, the optimal cutoff value for predicting ISR was 1.275 for IPI, yielding a sensitivity of 84.4% and specificity of 74.5% (area under the curve [AUC] = 0.872; 95% CI: 0.842–0.902; *p* < 0.001). The optimal cutoff value for NLR was 1.930 with a sensitivity of 78.0% and specificity of 78.8% (AUC = 0.784; 95% CI: 0.740–0.828; *p* < 0.001). For CAR, the best cutoff value was 0.700, corresponding to a sensitivity of 82.6% and specificity of 70.1% (AUC = 0.843; 95% CI: 0.813–0.874; *p* < 0.001) (Figure 1).

## 4. Discussion

In this study, IPI values were significantly higher in patients who developed in-stent restenosis (ISR) compared to those without ISR. This finding suggests that elevated IPI may serve as a potential biomarker for predicting ISR following DES implantation.

Inflammation is recognized as a fundamental pathophysiological mechanism in the initiation and progression of the atherosclerotic process. The pivotal role of inflammation in atherosclerosis was strongly demonstrated in a large-scale meta-analysis by Chen et al., which included 11.6 million genetic variants. That study showed that inflammation, in interaction with dyslipidemia, calcification, and adiposity, influences all stages of atherosclerotic development. Similarly, Runjic et al. reported that inflammatory markers and immunologic factors are significantly correlated with the severity of coronary artery disease (CAD). The persistent activity of chronic inflammation throughout all stages of atherosclerosis may explain the poorer cardiovascular outcomes observed in individuals with high inflammatory activity [32,33].

The pathophysiology of ISR involves endothelial injury, inflammatory response, and smooth muscle cell proliferation following stent implantation. Disruption of endothelial integrity after stent placement leads to platelet activation and the release of inflammatory mediators, which in turn promote smooth muscle cell migration into the intima, resulting in neointimal hyperplasia. Neutrophil-derived cytokines and proteolytic enzymes exacerbate endothelial injury and inflammation, whereas lymphocytes exert a regulatory role in modulating this process [34]. These mechanisms cause luminal narrowing, and in later stages, neoatherosclerosis and chronic inflammation further contribute to ISR development [35,36,37].

Although several mechanisms have been proposed to explain ISR, increasing evidence highlights the central role of an exaggerated inflammatory response in its pathogenesis. Recent studies have demonstrated strong associations between inflammation-based indices derived from routine blood parameters and ISR development. In patients with CAD, elevated neutrophil counts and decreased lymphocyte levels have been linked to adverse clinical outcomes, reinforcing the prognostic value of the neutrophil-to-lymphocyte ratio (NLR) as a predictor of restenosis [38,39,40]. Wang et al. reported that elevated NLR predicts ISR development in type 2 diabetic patients, while Siahaan et al., in a meta-analysis, confirmed that NLR has prognostic value for predicting ISR in both coronary and noncoronary stents [41].

Similarly, studies evaluating the glucose-to-lymphocyte ratio (GLR) have demonstrated that lower lymphocyte counts are significantly associated with the severity of CAD, indicating that lymphocytes play a crucial role in regulating the inflammatory response and influencing atherosclerotic progression [42]. C-reactive protein (CRP), a hepatic acute-phase protein, is a well-established biomarker for assessing inflammatory activity, and elevated CRP levels have been associated with endothelial dysfunction and vulnerable coronary plaques [43]. Conversely, serum albumin levels decline during inflammation, functioning as a negative acute-phase reactant. Hypoalbuminemia has been associated with decreased antioxidant, anti-inflammatory, and antiplatelet activity, and has shown an inverse relationship with ISR [44,45,46,47]. In this context, the CRP-to-albumin ratio (CAR) has been extensively studied as a strong marker of systemic inflammation.

Aksu et al. reported that CAR is an independent predictor of ISR in STEMI patients; Li et al. demonstrated that elevated CAR levels are associated with adverse five-year clinical outcomes in diabetic patients undergoing PCI; and Menekşe et al. found that CAR could predict in-hospital mortality in NSTEMI patients treated with PCI [48,49,50]. Based on these significant inflammatory parameters, composite indices reflecting a more comprehensive inflammatory response have been developed. The Systemic Immune-Inflammation Index (SII), calculated from neutrophil, lymphocyte, and platelet counts, has been found to be independently associated with ISR and to enhance prognostic accuracy [51]. Similarly, the Systemic Inflammation Response Index (SIRI), based on neutrophil, monocyte, and lymphocyte counts, has also been identified as a useful predictor of ISR [52].

In our study, higher CAR and NLR values were observed in patients with ISR, consistent with previous findings. This supports the concept that hematologic parameters and composite indices reflecting inflammatory activity may have potential clinical value in evaluating ISR risk after PCI. Despite major diagnostic and therapeutic advances, ISR-related recurrent cardiovascular diseases (CVDs) remain a major global health problem, prompting clinicians to intensify efforts to identify reliable predictive markers [53]. Although the role of IPI in predicting various cardiovascular conditions has been investigated in only a limited number of recent studies, its prognostic significance is increasingly recognized [54,55].

Saylık et al. demonstrated that IPI provides greater predictive accuracy for the no-reflow phenomenon compared with NLR and CAR in STEMI patients undergoing primary PCI [28]. Similarly, Jiang et al. recently reported that IPI is strongly associated with adverse clinical outcomes and can independently predict postprocedural complications such as contrast-induced nephropathy, severe arrhythmias, and myocardial infarction [56]. Yang et al. found that elevated IPI levels in heart failure patients were significantly associated with six-month mortality and offered superior prognostic performance compared to indices based solely on leukocyte subsets such as SII or SIRI [57]. Likewise, Oflar et al. showed that IPI is a useful parameter for predicting major adverse cardiac and cerebrovascular events (MACCE) in NSTEMI patients undergoing PCI [58].

The findings of our study indicate a potential relationship between IPI and ISR risk. This suggests that IPI may serve as an independent biomarker for predicting ISR and that the inflammatory burden at hospital admission could provide clinically relevant information regarding the risk of post-PCI complications.

IPI is a recently defined indicator that reflects both inflammatory and nutritional status. Its calculation integrates the CRP-to-albumin ratio (CAR) and the neutrophil-to-lymphocyte ratio (NLR). Each component of the IPI reflects fundamental inflammatory and metabolic pathways involved in ISR pathogenesis. CRP serves as a robust indicator of systemic inflammation, triggering vascular endothelial injury, cytokine release, and smooth muscle proliferation. Conversely, low albumin levels are associated with diminished antioxidant capacity, endothelial dysfunction, and increased inflammatory load. Neutrophils promote neointimal proliferation through the secretion of myeloperoxidase and proteolytic enzymes, whereas lymphocytes exert anti-inflammatory regulatory effects. Therefore, an elevated NLR reflects a predominance of proinflammatory activity over lymphocyte-mediated immune regulation [59,60,61,62].

Unlike single-parameter indices, IPI simultaneously captures the balance between proinflammatory and anti-inflammatory responses, offering a broader and more sensitive representation of systemic inflammation. Moreover, its simplicity, cost-effectiveness, and ease of calculation make IPI highly suitable for integration into clinical risk stratification models. Early identification of high-risk patients based on IPI may facilitate timely interventions, potentially preventing ISR and improving the clinical course of coronary artery disease. From a clinical standpoint, several strategies have been shown to reduce the risk of in-stent restenosis. Intravascular imaging-guided PCI (IVUS or OCT) improves stent expansion and reduces target lesion failure compared with angiography-guided procedures, as demonstrated in randomized trials such as ULTIMATE and IVUS-XPL [63,64] The use of contemporary new-generation DES with thinner struts and biocompatible or biodegradable polymers has also significantly lowered restenosis rates compared with earlier platforms [65,66]. Moreover, intensive secondary prevention—including high-intensity statin therapy, strict glycemic control, smoking cessation, and optimal blood pressure management—has been associated with reduced neointimal proliferation and improved long-term PCI outcomes [67]. In selected cases of ISR, drug-coated balloons represent an effective treatment strategy and have shown favorable outcomes in randomized trials [68]. Therefore, both procedural optimization and comprehensive cardiovascular risk factor control remain essential to minimize ISR development.

Future studies combining IPI and similar hematologic markers with advanced imaging modalities could enhance diagnostic precision and prognostic assessment. However, large-scale, multicenter, and prospective studies are required to confirm these findings and validate the clinical applicability of IPI.

## 5. Conclusions

This study demonstrates a significant association between IPI and the development of ISR in patients undergoing PCI. IPI, as an integrative marker reflecting systemic inflammatory and immunometabolic status, may serve as a practical and reliable prognostic biomarker in clinical practice.

### Study Limitations

This study has several limitations. First, the retrospective design carries an inherent risk of selection bias and the influence of uncontrolled confounders. Additionally, as the data were obtained from existing medical records, some clinical parameters may have been missing or inconsistently documented. Second, being a single-center study limits the generalizability of the findings to broader and more heterogeneous populations, as demographic, geographic, and healthcare differences may affect ISR development. Third, the analysis assessed only baseline IPI levels at admission and did not account for longitudinal changes or their impact on clinical outcomes. Genetic factors, lifestyle behaviors, and concomitant medications were also not evaluated, representing potential confounding variables. Fourth, again due to the retrospective design, longitudinal data on the control of conventional atherosclerotic risk factors (e.g., HbA1c, lipid levels, renal function progression) were not consistently available and therefore could not be included in the analysis. Furthermore, although our data on DAPT use in the first 12 months were sufficient, detailed data on the exact DAPT duration and adherence after 12 months were not consistently available. Lastly, due to its observational and retrospective nature, this study cannot definitively establish a causal relationship between IPI and ISR. These limitations highlight the need for cautious interpretation of the results and underscore the necessity of future prospective multicenter research to further elucidate the role of IPI in ISR development.

## Figures and Tables

**Table 1:** Baseline demographic and clinical characteristics of the study population.

Variables	ISR (−) (*n* = 353)	ISR (+) (*n* = 218)	*p* Value
Age, years	62 (55–68)	61 (52–68)	0.55
Male sex, *n* (%)	272 (77.1%)	165 (75.7%)	0.708
BMİ (kg/m^2^)	29.06 ± 2.56	28.9 ± 2.66	0.59
Hypertension, *n* (%)	178 (50.4%)	130 (59.6%)	0.032
Diabetes mellitus, *n* (%)	111 (34.7%)	81 (37.2%)	0.16
Heart failure, *n* (%)	45 (12.7%)	37 (17%)	0.162
Atrial fibrillation, *n* (%)	11 (3.1%)	6 (2.8%)	0.804
Cerebrovascular event, *n* (%)	4 (1.1%)	7 (3.2%)	0.08
COPD/Asthma, *n* (%)	10 (2.8%)	10 (4.6%)	0.271
Active smoking, *n* (%)	57 (16.1%)	52 (23.9%)	0.023
LAD lesion, *n* (%)	171 (58.4%)	107 (49.1%)	0.882
LCx lesion, *n* (%)	52 (14.7%)	23 (10.6%)	0.151
RCA lesion, *n* (%)	87 (24.6%)	70 (32.1%)	0.052
Bifurcation lesion	31 (8.8%)	16 (7.3%)	0.54
Multivessel Disease	127 (35%)	91 (42%)	0.085
Stent diameter (mm)	2.98 ± 0.61	3.1 ± 0.29	0.621
Stent length (mm)	23.5 ± 11.5	28.6 ± 14.3	0.061
LMCA lesion, *n* (%)	4 (1.1%)	5 (2.3%)	0.276
Beta-blocker use, *n* (%)	220 (62.3%)	141 (64.7%)	0.571
Calcium channel blocker use, *n* (%)	65 (18.4%)	47 (21.6%)	0.358
ACEI/ARB use, *n* (%)	177 (50.1%)	117 (53.7%)	0.412
Statin use, *n* (%)	242 (68.6%)	153 (70.2%)	0.682
Aspirin use, *n* (%)	282 (79.9%)	174 (79.8%)	0.984
P2Y12 inhibitor use, *n* (%)	113 (37.2%)	87 (39.9%)	0.055

Abbreviations: BMİ—body mass index; ISR—in-stent restenosis; COPD—chronic obstructive pulmonary disease; LAD—left anterior descending artery; LCx—left circumflex artery; RCA—right coronary artery; LMCA—left main coronary artery; ACEI—angiotensin-converting enzyme inhibitor; ARB—angiotensin receptor blocker.

**Table 2:** Comparison of laboratory parameters between patients with and without in-stent restenosis (ISR).

Variables	ISR (−) (*n* = 353)	ISR (+) (*n* = 218)	*p*-Value
Glucose (mg/dL)	119.3 ± 36	128.78 ± 18.4	<0.001
Creatinine (mg/dL)	1 ± 0.43	1.01 ± 0.35	0.678
GFR (mL/min/1.73 m^2^)	78.84 ± 19.83	77.81 ± 21.4	0.587
Calcium (mg/dL)	9.4 (9.05–9.7)	9.32 (9.1–9.6)	0.767
Potassium (mmol/L)	4.5 (4.2–4.78)	4.52 (4.12–4.9)	0.706
Sodium (mmol/L)	138.67 ± 3.54	138.37 ± 3.16	0.3
Albumin (g/dL)	4.42 ± 0.2	4.09 ± 0.5	0.02
AST (U/L)	25.02 ± 15.7	27.35 ± 11.2	0.058
ALT (U/L)	23.62 ± 14.14	25.68 ± 13.97	0.09
WBC (×10^9^/L)	8.71 ± 1.6	8.96 ± 2.5	0.157
Monocyte (×10^9^/L)	0.64 ± 0.23	0.71 ± 0.31	0.007
Neutrophil (×10^9^/L)	4.48 ± 1.09	6.39 ± 2.96	<0.001
Lymphocyte (×10^9^/L)	2.79 ± 0.8	2.59 ± 1.05	0.01
Platelet (×10^9^/L)	246.83 ± 75.9	258.63 ± 64.35	0.057
Eosinophil (×10^9^/L)	0.172 ± 0.124	0.168 ± 0.121	0.67
LDL-C (mg/dL)	103.99 ± 28.24	111.04 ± 66.78	0.081
HDL-C (mg/dL)	42.42 ± 10.79	41.63 ± 10.59	0.395
Total cholesterol (mg/dL)	175.6 ± 43.23	179.068 ± 41.68	0.346
Triglycerides (mg/dL)	177.09 ± 92.7	183.67 ± 100.71	0.426
CRP (mg/L)	4.07 ± 3.83	6.1 ± 4.18	<0.001
CAR (CRP/Albumin ratio)	0.41 (0.24–0.81)	1.26 (0.71–2.13)	<0.001
NLR	1.62 (1.38–1.86)	2.5 (2.04–3.1)	<0.001
IPI	0.71 (0.38–1.37)	3.13 (1.67–6.5)	<0.001

Abbreviations: ISR, in-stent restenosis; GFR, glomerular filtration rate; AST, aspartate aminotransferase; ALT, alanine aminotransferase; WBC, white blood cell; LDL-C, low-density lipoprotein cholesterol; HDL-C, high-density lipoprotein cholesterol; CRP, C-reactive protein; CAR, CRP-to-albumin ratio; NLR, neutrophil-to-lymphocyte ratio; IPI, inflammatory prognostic index.

**Table 3:** Univariate and multivariate logistic regression analyses identifying independent predictors of in-stent restenosis.

	Univariate Analysis			Multivariate Analysis		
	Odds Ratio	95%CI	*p* Value	Odds Ratio	95%CI	*p* Value
Glucose	1.01	1.004–1.016	0.001	1.007	1.001–1.014	0.032
Hypertension	0.639	0.489–0.969	0.032	0.72	0.454–1.142	0.163
Active smoking	1.627	1.068–2.479	0.024	2.135	1.236–3.687	0.007
Albumin *	0.313	0.21–0.467	<0.001	0.465	0.299–0.724	0.001
CRP *	1.137	1.086–1.19	<0.001	1.087	1.031–1.145	0.002
Neutrophil *	1.694	1.497–1.916	<0.001	1.878	1.593–2.213	<0.001
Lymphocyte *	0.782	0.647–0.944	0.011	0.593	0.437–0.803	0.001
Monocyte	2.36	1.248–4.465	0.008	0.375	0.146–0.967	0.042
CAR *	6.81	4.745–9.774	<0.001	6.429	4.176–9.899	<0.001
NLR *	3.81	2.85–5.09	<0.001	3.392	2.516–4.574	<0.001
IPI *	2.741	2.55–3.331	<0.001	2.898	2.351–3.573	<0.001

Abbreviations: CI—confidence interval; CRP—C-reactive protein; CAR—C-reactive protein to albumin ratio; NLR—neutrophil-to-lymphocyte ratio; IPI—inflammatory prognostic index. * Significant variables included in multivariate analysis.

## Data Availability

The data presented in this study are available on reasonable request from the corresponding author. The data are not publicly available due to privacy and ethical restrictions, as they contain patient-level clinical information obtained from hospital medical records.
